# Occupational Balance in Refugees: The Role of Well-Being, Participation, and Perceived Discrimination

**DOI:** 10.3390/ijerph22071077

**Published:** 2025-07-06

**Authors:** Kardelen Yıldırım, Gülşah Zengin Yazıcı, Beyzanur Demirci, Sümeyye Sarışahin, Sedef Şahin

**Affiliations:** 1 Department of Occupational Therapy, Faculty of Health Sciences, Bezmialem Vakif University, 34065 Istanbul, Turkey; beyzanur.demirci@bezmialem.edu.tr (B.D.); sumeyye.sarisahin@bezmialem.edu.tr (S.S.); 2 Institute of Health Sciences, Faculty of Health Sciences, Department of Occupational Therapy, Bezmialem Vakif University, 34065 Istanbul, Turkey; gulsah.zengin@bezmialem.edu.tr; 3 Department of Occupational Therapy, Faculty of Health Sciences, Hacettepe University, 06230 Ankara, Turkey; skarayazgan@hacettepe.edu.tr

**Keywords:** discrimination, occupational balance, participation, refugees, Syrian people, well-being

## Abstract

Although the experiences of forced migration among refugees have been widely studied, the relationships between occupational balance, well-being, participation, and perceived discrimination remain poorly understood. This study aimed to examine these interrelationships and to explore the predictive role of occupational balance in each domain. Data were collected between February and March 2025 from 260 Syrian refugees aged 18–65 using validated instruments: the Occupational Balance Questionnaire, Personal Well-Being Index–Adult, Participation Scale, and Perceived Discrimination Scale. Occupational balance was significantly correlated with well-being, participation, and perceived discrimination (all *p* < 0.001). However, regression analyses revealed that only well-being (β = 0.114, *p* < 0.001) and participation (β = −0.107, *p* = 0.002) significantly predicted occupational balance; perceived discrimination had no direct effect. These findings highlight the critical role of occupational balance in fostering psychosocial integration. Enhancing refugees’ well-being and participation may support their adaptation processes. Community-based, culturally responsive interventions that target these domains could promote social inclusion, continuity of roles, and long-term psychosocial stability among displaced populations in host societies.

## 1. Introduction

Forced migration is a complex phenomenon with far-reaching consequences for individuals, encompassing not only physical displacement but also economic, social, political, and security-related challenges [1]. As of June 2024, approximately 122.6 million people worldwide were forcibly displaced, including around 43.7 million recognized as refugees [2]. The Syrian civil war, widely recognized as the most severe humanitarian crisis since World War II, has displaced over six million people, making Syrians the largest refugee population worldwide [3]. Turkey, as a key host country, currently accommodates 2.9 million refugees under temporary protection, according to official data [4]. Migration trajectories of individuals seeking new lives demonstrate that the effects of forced migration extend beyond political boundaries and policy frameworks [5]. With rising ethno-cultural diversity driven by migration, there is a pressing need for contemporary research on assimilation and integration processes to better understand evolving community dynamics, including the complex interrelations among racism, identity formation, and social change [6]. Consequently, scholarly efforts must focus on the factors that shape refugees’ health and everyday life experiences to gain a comprehensive understanding of their lived realities [7].

### 1.1. Occupational Balance

Occupational balance, a key indicator of health, refers to the harmony and variety among meaningful and purposeful activities that reflect the voluntary, responsible, and significant use of time across different life domains [8]. It extends beyond simply allocating equal or proportional time to various activities [9], encompassing alignment with personal values, a sense of autonomy, balance among life roles, and overall well-being [10]. Key factors shaping occupational balance include managing multiple roles (such as parent, employee, or student), engaging in intrinsically motivating occupations, and accessing environmental resources [11]. In contexts characterized by structural inequalities, such as forced migration, social exclusion and discrimination significantly disrupt occupational balance [12].

Forcibly displaced individuals often face occupational disruption, characterized by exclusion from meaningful activities due to temporal, physical, and sociocultural barriers [13]. This disruption often leads to occupational dysfunction, a condition arising when activities and tasks are not integrated coherently or satisfactorily, thereby hindering participation in daily activities [14]. Occupational balance is widely recognized as an important determinant of enhanced health, well-being, and life satisfaction [15]. The perceived value of life roles closely influences occupational balance; consequently, restrictions in participation and imbalances in daily activities negatively impact overall health and well-being [16].

Occupational therapy, a discipline oriented toward individuals, groups, and communities, examines relationships between individuals and their engagement in meaningful daily activities. Guided by principles of participation, inclusion, and adaptation, occupational therapy explores how people navigate disruptions to routines, roles, and environments, particularly in conditions of independence challenges, displacement, marginalization, or social exclusion [17]. Recently, occupational therapy’s scope has significantly expanded beyond traditional clinical settings, increasingly encompassing diverse populations, including refugees [18]. This expansion has shifted the field’s focus toward addressing everyday challenges experienced by these populations, who strive to reconstruct daily lives and social identities within unfamiliar environments [19].

### 1.2. Well-Being

The World Health Organization defines health not merely as a biological condition but as a holistic state of well-being that includes life satisfaction, a sense of meaning, and harmony with one’s social environment [20]. Well-being is a multidimensional state involving mental, social, physical, and spiritual aspects of an individual’s life, participation in activities, and supportive personal contexts [21]. According to Bennet et al. [22], refugees who resettle in new countries often experience major life transitions—including role changes, identity shifts, and professional disruptions—which can interfere with daily occupations and negatively affect overall well-being. Forced migration and resettlement are commonly associated with reduced well-being, largely due to a loss of control over personal circumstances, which undermines refugees’ sense of identity, purpose, and future orientation [23]. Yalim’s study [24] demonstrated that the well-being of Syrian refugees is closely linked to their living conditions, with factors such as financial hardship, limited social support, discrimination, and legal status exerting a direct impact on their mental health. Examining well-being is thus crucial for understanding the daily challenges refugees face, providing deeper insights into their subjective experiences that extend beyond objective measures like quality of life [22]. Establishing meaningful balance among various daily activities directly contributes to improved health, enhanced participation, and overall well-being [25].

### 1.3. Participation

Refugees often experience major disruptions to their daily routines following prolonged periods of deprivation and may face restrictions in participation [26,27]. Participation refers to individuals actively engaging in daily activities they find meaningful and valuable [28]. Assessing participation helps individuals identify their occupational priorities and the types of support they need [29]. Active societal involvement and cultural belonging significantly contribute to identity formation through occupational engagement [30]. Occupational balance plays a critical role in promoting participation among refugees, closely relating to occupational disruption and deprivation—both common outcomes of forced migration [31]. Additionally, refugees’ everyday experiences depend not only on personal factors but also on dynamic interactions among their social environment, cultural identity, and migration-related transitions [32]. Limited leisure opportunities and restricted social networks negatively affect refugees’ participation and social integration processes [33]. Research on immigrant populations has shown that social exclusion, which is often the result of factors such as unemployment, poverty, poor health, discrimination, and limited participation, highlights the importance of examining these interconnected dimensions within refugee contexts [34].

### 1.4. Perceived Discrimination

Social exclusion, often rooted in experiences of discrimination, refers to the marginalization of certain individuals or groups who are affected by certain conditions or events [35]. The perception of general discrimination emerges when individuals believe they are subject to widespread societal bias. This perception can be categorized into two types: group discrimination, which involves the systemic exclusion of a particular community, and personal discrimination, where the individual is directly targeted [36]. Discrimination itself represents a multidimensional phenomenon occurring at both group and individual level, either directly and expressed (blatant) or indirectly, implicitly and often difficult to recognize (subtle) [37]. Refugees often face social exclusion due to a range of factors, including inadequate living arrangements, limited education and language skills, unemployment, and challenges related to settlement and relocation, all of which contribute to their marginalization in the host country [38]. Studies have shown significant associations between perceived discrimination and negative outcomes among refugees, such as declining mental health [39], reduced social participation and community integration [33], and lower levels of subjective well-being [40]. All forms of discrimination stigmatize refugees, undermining their self-esteem and self-efficacy, damaging their relationships with others and institutions, and restricting their access to resources, development opportunities, and pathways to social integration [41]. Goffman’s concept of stigma explains how individuals labeled as refugees experience exclusion that prevents their integration into society and leads to marginalization from broader social groups [12]. Social isolation is a critical concern for refugee populations, who frequently lack access to social networks and language proficiency, thereby impeding their ability to perform parental, professional, and social roles effectively [42]. These challenges underscore the need to develop equitable and accessible health services and social policies that address the needs of both refugees and host communities [43].

### 1.5. Theoretical Background

This study adopts the Occupational Justice Framework, which asserts that individuals possess the right to engage in occupations they find meaningful and beneficial for themselves, their families, communities, and societies, as such activities foster well-being, identity, and social participation [44]. Within this framework, structural inequalities, including forced migration, marginalization, and discrimination, may limit access to meaningful occupations, disrupting both occupational balance and participation [45]. The framework conceptualizes well-being and participation as indicators of occupational inclusion and adaptation, while it regards perceived discrimination as a manifestation of occupational injustice [46]. Together, these constructs offer a theoretically grounded perspective for understanding the daily experiences of refugees and their adaptation processes within host societies [47].

Occupational balance also reflects an effort to create a coherent daily life structure from a societal perspective, highlighting the importance of examining the occupational balance of refugees and the factors that influence it [48]. Recent studies increasingly identify well-being, participation, and perceived discrimination as key factors shaping refugees’ occupational experiences and integration processes [49]. Although interest in refugee health has grown, previous studies have seldom explored the combined effects of well-being, participation, and perceived discrimination on occupational balance. A recent literature review found no research explicitly investigating this tripartite relationship within refugee populations, revealing a notable gap in the literature. The primary aim of this study is to examine the relationship between occupational balance, well-being, participation, and perceived discrimination among refugees. The secondary aim is to investigate the predictive effect of refugees’ occupational balance on well-being, participation, and perceived discrimination. In this context, the hypotheses of the study are as follows:

1. There is a relationship between refugees’ occupational balance, well-being, participation, and their perceived discrimination levels.
2. Well-being, participation and discrimination are significant predictors of refugee’ occupational balance.

## 2. Materials and Methods

### 2.1. Design

This study used a cross-sectional research design. A purposive sampling method was applied to recruit refugees in line with the study’s objectives. Istanbul, which hosts the largest refugee population in Turkey, served as the research site [4]. In this context, researchers contacted non-governmental organizations (NGOs) in Istanbul that provide legal, socio-economic, and psychosocial support to refugees and other migrants. These NGOs, established to facilitate the social integration of refugees and asylum seekers under international humanitarian law, supported the recruitment process. With assistance from NGO staff and announcements made in the organizations’ waiting areas, researchers reached refugees and their family members. All potential participants received detailed information about the study from the researchers. Those who agreed to participate provided written informed consent and completed the study questionnaires in a quiet and appropriate setting.

### 2.2. Settings and Participants

Participants consisted of adult refugees who had applied to NGOs in Istanbul and resided either in the city center or in communal living areas within the city. The data collection period extended from February 2025 to March 2025.

Participants had to meet the following criteria to be included in the study: (i) be between the ages of 18–65, (ii) be of Syrian origin, (iii) be able to read and write Turkish (literate), (iv) have lived in Turkey for at least 5 years, and (v) have been living under temporary protection in Turkey. Participants were excluded if they met the following criteria: (i) have a known physical, neurological, or developmental disorder, (ii) cannot communicate fluently and (iii) not being willing to participate in the study.

Researchers recruited participants through two NGOs that provided services to refugees between February–March 2025. All participants held official refugee status. Although all participants reported Arabic as their native language, Turkish literacy was a requirement for participation. Researchers informed all potential participants about the purpose and procedures of the study, emphasizing the voluntary nature of participation. Written informed consent was obtained from those who agreed to take part, and participants were reminded that they could withdraw from the study at any point. Researchers ensured participants had sufficient time to complete the study instruments and provided explanations when necessary. All interviews took place in quiet, calm, and safe rooms within NGO facilities, specifically selected to minimize distractions and ensure participant comfort. Before starting the study, the sample size was calculated using the G*Power 3.1. 9.6 program, and a sample calculation was made for correlation. In the calculation, the effect size was 0.2 (d = 0.2), the margin of error was 5% (α = 0.05), and the power was 90% (1-β = 0.90), and the sample size was determined as 258 [50].

**Figure 1:**
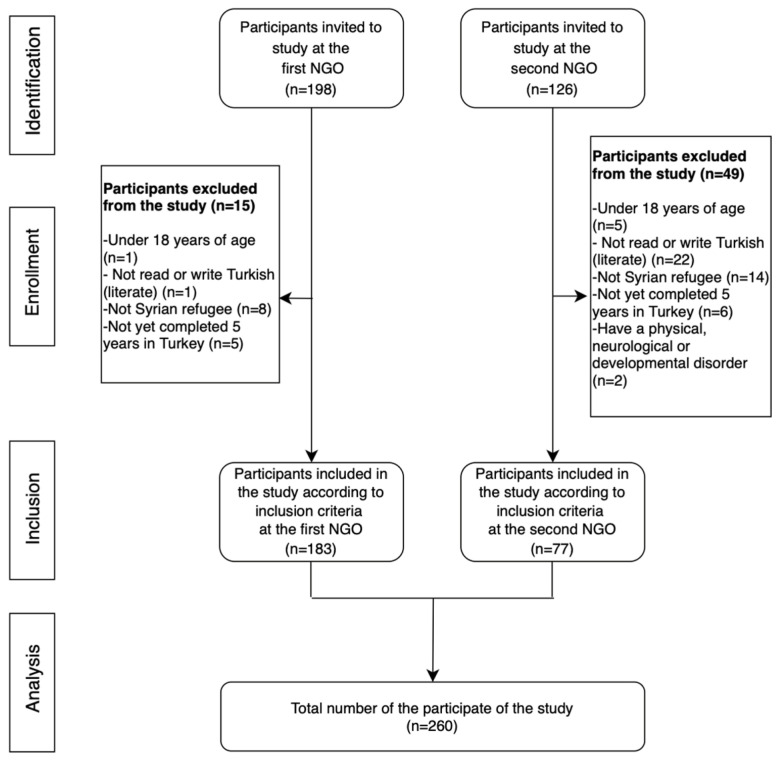
Flow chart. Note. NGO: Non-governmental organization.

A total of 324 participants were invited within the sample size of the study, and 64 people were excluded from the study according to predetermined criteria. The number of participants participating in the study was finally determined as 260. The data collection process is shown in Figure 1.

### 2.3. Data Collection Tools

#### 2.3.1. Sociodemographic Information Form

The Sociodemographic Information Form, developed by the researchers, included questions to collect detailed background information from the participants. Specifically, participants were asked to report their age, gender (e.g., female or male), educational level (e.g., primary school, secondary school, high school, university, master’s, or doctorate), employment status (e.g., full-time, part-time, temporary, or unemployed), and marital status (e.g., single, married, divorced, widowed).

#### 2.3.2. Occupational Balance Questionnaire (Revised Version, OBQ11)

The OBQ11 measures individuals’ satisfaction with the amount and variety of their daily activities and defines their occupational balance based on the obtained results [51]. The scale includes items such as “*In an ordinary week, I feel that I have enough to do*” and “*There is a balance between what I do for myself and what I do for others*”, offering a comprehensive assessment of perceived balance across different life domains. It consists of 11 items rated on a 4-point Likert scale ranging from 0 (strongly disagree) to 3 (strongly agree). Total scores range from 0 to 33, with higher scores indicating greater occupational balance. Günal et al. [52] conducted the Turkish adaptation of the OBQ11. The adaptation process followed internationally recognized translation and back-translation procedures, complemented by expert panel reviews and pilot testing to ensure cultural and linguistic appropriateness for Turkish populations. Construct validity analysis confirmed the unidimensional structure of the scale, with factor loadings ranging from 0.58 to 0.84. Model fit indices demonstrated acceptable levels, with RMSEA at 0.061, CFI at 0.92, and TLI at 0.91. Internal consistency reliability yielded a Cronbach’s alpha of 0.78, while test–retest reliability produced an intraclass correlation coefficient (ICC) of 0.92.

#### 2.3.3. Personal Well-Being Index-Adult (PWI-A)

The PWI-A measures subjective well-being by capturing individuals’ satisfaction across various life domains, in alignment with the conceptual structure of well-being. Items such as “*How satisfied are you with your health?*” and “*How satisfied are you with feeling part of your community?*” reflect participants’ subjective evaluations. The instrument consists of eight items covering standard of living, personal health, life achievements, personal relationships, personal security, community belonging, future security, and spirituality or religion. Participants rate their satisfaction in each domain on a scale from 0 (no satisfaction at all) to 10 (completely satisfied) [53]. The lowest possible score on the scale is 0, while the highest is 80. The score obtained from the scale corresponds to the average of the eight sub-areas, and the increase in the score corresponds to the increase in the perception of personal well-being. The meaning of the relevant score is determined by the calculation of [(Total Score Obtained from the Scale/Highest Score Obtained from the Scale) × 100] [54]. Meral [55] conducted the Turkish adaptation and validation of the PWI-A. Construct validity was evaluated using exploratory and confirmatory factor analyses. The CFA results supported the original eight-domain structure with satisfactory fit indices (CFI = 0.91, RMSEA = 0.07). Convergent validity was established through correlations with the WHOQOL-BREF domains (r values ranged from 0.41 to 0.63, *p* < 0.01). The internal consistency was high (Cronbach’s α = 0.86).

#### 2.3.4. Participation Scale (PS)

The PS evaluates perceived restrictions across key socioeconomic life areas and was used in this study to assess individuals’ levels of participation [56]. Items such as “*Do you have equal opportunities as your peers to find a job?*” and “*Do you participate in important celebrations or invitations such as weddings, funerals, or religious events?*”, capture the extent of participation restrictions across various domains. The questionnaire includes questions related to learning and applying knowledge and skills, general tasks and demands, communication, mobility, self-care, domestic life, interpersonal interactions and relationships, major life areas, and community, social, and civic life. The PS employs a 5-point rating system where responses are scored as 0 (no significant restriction), 1 (mild restriction), 2 (moderate restriction), 3 (severe restriction), and 5 (extreme restriction). The scale includes 18 items, yielding a total score ranging from 0 to 90. Interpretation of the total score follows these ranges: 0–12 indicates no participation restriction, 13–22 indicates mild restriction, 23–32 indicates moderate restriction, 33–52 indicates severe restriction, and 53–90 indicates very severe restriction. The final score reflects the overall level of participation restriction. Altuntaş et al. [57] conducted the Turkish adaptation of the PS. Construct validity was examined using both exploratory factor analysis (EFA) and confirmatory factor analysis (CFA), which confirmed a two-factor model consistent with the original structure. Model fit indices were within acceptable ranges (RMSEA = 0.067, CFI = 0.93, TLI = 0.91). The scale showed strong internal consistency (Cronbach’s α = 0.85) and excellent composite reliability (McDonald’s ω = 0.92).

#### 2.3.5. Perceived Discrimination Scale (PDS)

The scale was developed by Ruggiero and Taylor in 1995 with the aim of measuring individuals’ perceptions of discrimination. The scale captures participants’ subjective experiences of discrimination directed toward their ethnic group within the host society. It consists of two subscales, group discrimination and personal discrimination, each comprising four items, resulting in a total of eight items. The group discrimination subscale evaluates perceived discrimination directed toward the participant’s ethnic group as a whole. Sample items include: “*To what extent do Syrians experience discrimination when looking for a job?*” and “*To what extent do Syrians experience discrimination when walking in the street or shopping*”. The personal discrimination subscale assesses participants’ personal experiences of exclusion and emotional discrimination. Sample items include the following: “*I do not feel accepted by the local people” and “Because I am Syrian, people have isolated me and did not allow me to join their groups”*. Responses are rated on a five-point scale ranging from 1 (never) to 5 (always) [36]. As the total score increases, the level of perceived discrimination also increases. Baysu [58] validated the Turkish version of the scale. Both exploratory and confirmatory factor analyses confirmed the two-factor structure, with acceptable model fit indices (RMSEA = 0.059, CFI = 0.95, SRMR = 0.045). Factor loadings ranged from 0.61 to 0.88. Criterion validity was supported by significant correlations with depression and anxiety measures. Güloğlu [59] reported high internal consistency among refugee samples, with Cronbach’s alpha values of 0.91 for the group subscale, 0.93 for the personal subscale, and 0.95 for the total score.

### 2.4. Data Analysis

Researchers conducted all statistical analyses using IBM SPSS Statistics (Version 26.0) and Python (Version 3.8). The analysis began with an assessment of the normality of the dependent variable. Descriptive statistics, skewness and kurtosis values, histograms, normal probability plots, and Q–Q plots were examined to evaluate the distribution. Multivariate normality assumptions were also reviewed prior to conducting further analyses.

Before performing the regression analysis, Pearson correlation analysis was used to explore the relationships between the dependent variable (OBQ11) and the independent variables, which included the Personal Well-Being Index, Participation Scale, Group Discrimination, Personal Discrimination, and Total Perceived Discrimination. This analysis identified the strength and direction of associations and assessed potential multicollinearity among variables. The strength of the correlations was evaluated according to Cohen’s effect size guidelines, with correlation coefficients (r) around 0.10 indicating a small effect, 0.30 indicating a medium effect, and 0.50 or higher indicating a large effect [60].

Following the correlation analysis, researchers performed a multiple linear regression analysis to evaluate the predictive effects of the independent variables on the OBQ11 total score. The Enter method was applied, allowing all independent variables to enter the model simultaneously. The significance of the overall model was tested using ANOVA, while the coefficient of determination (R^2^) assessed the explanatory power of the predictors. Standardized beta coefficients (β) were examined to determine the relative contribution of each independent variable. Based on Cohen’s guidelines, beta values around 0.10 reflected a small effect, values near 0.30 indicated a medium effect, and values of 0.50 or greater represented a large effect [60].

To validate the regression model, researchers tested key assumptions. Multicollinearity was evaluated through Variance Inflation Factor (VIF) values, while homoscedasticity and residual normality were assessed by examining standardized residual histograms and normal probability plots. All statistical tests were conducted at a significance level of *p* < 0.05.

### 2.5. Ethical Considerations

The University Non-Interventional Research Ethics Board approved the study (Decision number: 61351342/020-798). Researchers conducted the study in accordance with the Declaration of Helsinki. Participants received detailed information outlining the study’s purpose, data collection procedures, and the voluntary nature of participation. Researchers assured participants that withdrawing from the study would not affect their employment status and that all collected data would be handled and analyzed in a manner that ensured confidentiality and protected participants’ integrity.

## 3. Results

The descriptive statistics of the participants are presented in Table 1. The sample consisted of 260 participants, of whom 48.85% were female and 51.15% were male. The majority had either a university degree (34.23%) or a high school education (34.23%). Regarding employment status, 44.23% were employed full-time, while 39.23% were unemployed. Marital status data revealed that 54.23% were married, and 37.30% were single. Participants’ ages ranged from 18 to 65 years, with a mean age of 32.54 years (SD = 1.48). Table 1 also summarizes the descriptive statistics for the Personal Well-Being Index, Participation Scale, Perceived Discrimination Scale, and OBQ11 scores.

**Figure 2:**
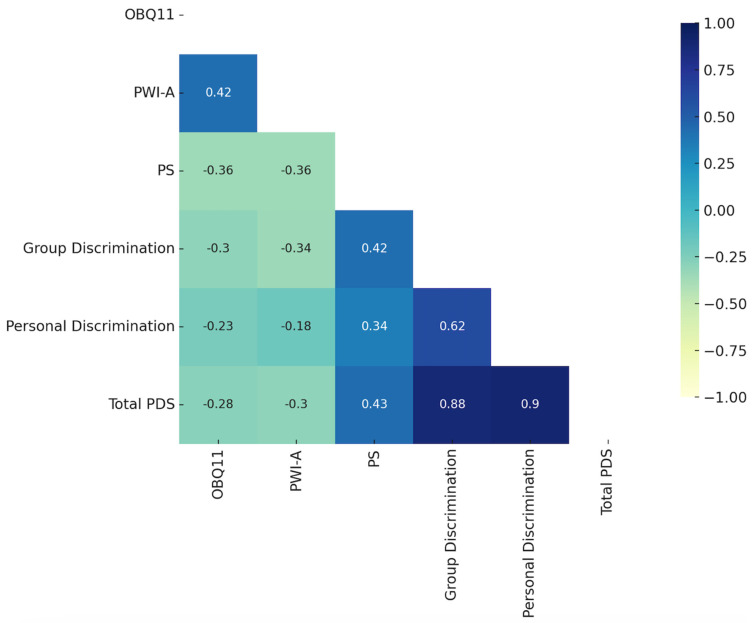
Pearson correlation matrix. Note. OBQ11: Occupational Balance Questionnaire; PWI-A: Personal Well-Being Index-Adult; PS: Participation Scale; PDS: Perceived Discrimination Scale.

The relationships among the study variables were examined through Pearson correlation analysis, with the results presented in Figure 2.

Pearson correlation analysis indicated a moderate positive and significant relationship between the total OBQ11 score and the Personal Well-Being Index (r = 0.421, *p* < 0.001). In contrast, the total OBQ11 score showed a moderate negative and significant correlation with Participation (r = −0.357, *p* < 0.001) and Group Discrimination (r = −0.303, *p* < 0.001). Additionally, weak negative correlations were observed between the total OBQ11 score and Personal Discrimination (r = −0.227, *p* = 0.002), as well as Total Perceived Discrimination (r = −0.284, *p* < 0.001) (see Table 2).

The regression model demonstrated moderate explanatory power, with an R value of 0.494, indicating a moderate relationship between the independent variables and the OBQ11 total score. The R^2^ value reached 0.244, suggesting that approximately 24.4% of the variance in OBQ11 scores was explained by the predictor variables included in the model. After adjusting for model complexity, the adjusted R^2^ value of 0.229 confirmed that the predictors still accounted for 22.9% of the variance. The standard error of the estimate, calculated as 4.971, reflected the average deviation of observed values from predicted values and indicated the model’s predictive accuracy. Results from the ANOVA showed that the overall regression model was statistically significant (F = 16.309, *p* < 0.001), demonstrating that the independent variables significantly contributed to predicting OBQ11 scores. These findings indicate that the model explains a meaningful portion of the variance in occupational balance.

The multiple linear regression analysis further revealed that the independent variables significantly predicted the total OBQ11 score (F (5,252) = 16.309, *p* < 0.001). Examining the regression coefficients, the Personal Well-Being Index had a moderate positive and significant effect on the total OBQ11 score (*β* = 0.323, t = 5.327, *p* < 0.001). The Participation Scale variable had a small but significant negative effect on the OBQ11 score (*β* = −0.197, t = −3.132, *p* = 0.002). While Group Discrimination and Personal Discrimination exhibited moderate negative effects, their significance levels were marginal (*p* = 0.080 and *p* = 0.105, respectively). Meanwhile, the Total Perceived Discrimination variable was not a statistically significant predictor (*p* = 0.146). The detailed regression results are provided in Table 3.

**Figure 3:**
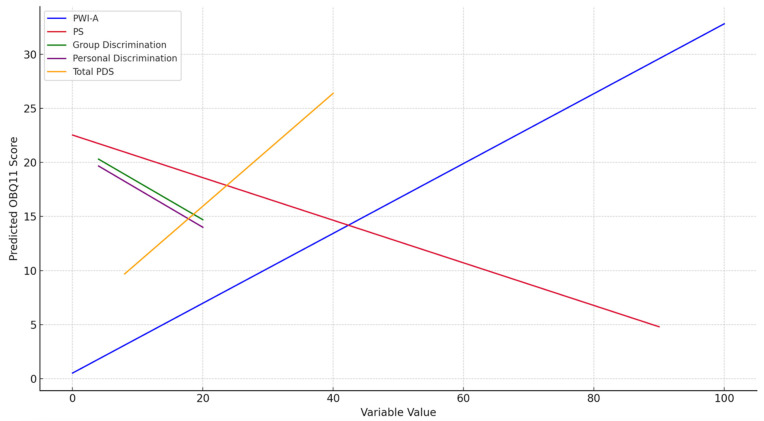
Predicted OBQ11 scores by independent variable levels based on the linear regression model. Note. OBQ11: Occupational Balance Questionnaire; PWI-A: Personal Well-Being Index-Adult; PS: Participation Scale; PDS: Perceived Discrimination Scale.

The linear regression model explained 24.4% of the variance in OBQ11, indicating a moderate explanatory power. Based on the standardized beta coefficients, Personal Well-Being Index had a significant positive effect, while Participation Scale had a significant negative effect on occupational balance. Although Total PDS shows a positive trend in the prediction plot, its effect was not statistically significant in the regression model (*p* = 0.146). The predictive trends of all variables included in the model are visually summarized in Figure 3.

Researchers conducted preliminary assumption tests to ensure the appropriateness of the regression analysis. Descriptive statistics, along with skewness and kurtosis values, were examined for each variable. All values fell within the acceptable range of ±1.5, indicating approximate normality. Visual inspections using histograms, Q–Q plots, and normal probability plots further supported the normal distribution of the data.

Multicollinearity was assessed using Variance Inflation Factor (VIF) values for each independent variable, all of which were well below the conventional threshold of 5, indicating no multicollinearity concerns. The VIF values were as follows: Personal Well-Being Index (VIF = 1.32); Participation Scale (VIF = 1.29); Group Discrimination (VIF = 1.25); Personal Discrimination (VIF = 1.27); Total Perceived Discrimination (VIF = 1.40).

Residual analyses demonstrated no major violations of assumptions. The standardized residuals approximated a normal distribution, and the scatterplot of residuals versus predicted values indicated homoscedasticity. These findings confirm that the assumptions of normality, linearity, homoscedasticity, and independence were met, validating the application of multiple linear regression.

## 4. Discussion

The findings of this study demonstrate a significant association between refugees’ occupational balance and their levels of well-being, participation, and perceived discrimination. Well-being and participation emerged as significant predictors of occupational balance, while perceived discrimination did not exert a direct effect. These results suggest that perceived discrimination may influence certain aspects of refugees’ daily life experiences without directly determining their occupational balance. To the best of our knowledge, this is the first study to explore the combined effects of well-being, participation, and perceived discrimination on the occupational balance of refugees.

Although the majority of participants in this study were male, the gender distribution was relatively balanced. This aligns with official data on the refugee population in Turkey, which reflects a similar pattern [4]. Concurrently, the majority of subjects in the study are refugees who have attained a high school diploma or university degree. While it is acknowledged that armed conflict and involuntary displacement can disrupt educational opportunities for numerous individuals, the imperative to guarantee the right to education for those under temporary protection is articulated in the 1951 Refugee Convention and the 1967 Protocol Relating to the Status of Refugees [61,62]. It is evident that the optimal educational outcomes are facilitated by simultaneous identification with both the host and home cultures. However, the cultural adaptation of refugees is influenced by educational attainment, school adjustment, attachment, academic achievement, and social support [62]. The fact that the refugees included in our study have been residing in Turkey for a minimum of five years may suggest that their social adaptation has improved. Consequently, there is a clear necessity for research that focuses more on the processes related to the social integration of individuals with higher education levels.

In our study, well-being emerged as the strongest predictor of occupational balance among refugee participants. This finding highlights the crucial role of psychological well-being in enabling displaced individuals to sustain meaningful and structured engagement in daily life. Refugees with higher levels of personal well-being may possess greater internal and external resources to navigate resettlement challenges and sustain occupational stability. This finding aligns with previous research highlighting the importance of occupational balance in improving psychosocial outcomes. For example, Belhan Çelik et al. [63] demonstrated that enhancing occupational balance through telerehabilitation positively influenced well-being among Syrian refugee children. Similarly, Darawsheh [64] argued that disruptions in occupational balance can lead to occupational deprivation and diminished well-being. McCarthy et al. [65] also stressed the importance of maintaining daily occupations and social networks to promote resilience and adjustment during forced migration. Our findings reinforce the view that interventions targeting refugee well-being must consider the occupational dimension as a central pillar. In this context, we advocate for individualized, community-based, and identity-sensitive occupational therapy approaches that address the intersecting challenges of displacement, loss, and resettlement. By way of illustration, a “Community Garden Project” could be developed in which small-scale urban gardens are co-designed and maintained under the guidance of occupational therapists; the volunteer-run “Leisure Garden” initiative for Syrian refugee children in İzmir, Turkey, can be cited as a precedent [66]. Projects of this kind emphasize the value of multidisciplinary, community-driven gardening interventions tailored to displaced populations. Moreover, gardening has been shown to support mental health, foster a sense of belonging and solidarity, diversify livelihood opportunities, and enhance food security and dietary quality among displaced communities [67].

This study found a significant relationship between occupational balance and participation among refugees, identifying occupational balance as a key predictor of participation. By empirically demonstrating this link, the study adds new evidence to the growing research on migration, well-being, and social inclusion. Håkansson and Wagman [68], for example, showed that occupational balance increases individuals’ participation in daily life, supporting both physical and psychological well-being. Similarly, Yazdani et al. [69] noted that occupational therapists define occupational balance as a factor that enables individuals to engage in meaningful activities. Occupational balance as a necessity for individuals to adapt to their life roles and suggested that its disruption could lead to a lack of participation [16]. Our findings highlight the relevance of these insights for refugee integration policies, especially regarding employment and psychosocial support services. Promoting occupational balance should serve as a strategic objective in policy design aimed at increasing participation. Although participation emerged as a statistically significant predictor of occupational balance, its effect size was relatively modest. This suggests that participation contributes meaningfully to the model but does not explain occupational balance on its own. Future research could explore potential mediators or moderators, such as social support, trauma history, or resilience, that could amplify the role of participation in shaping occupational balance. Occupational therapy interventions for refugee populations should move beyond individual skill-building to also address structural and environmental barriers. These include limited access to employment, insecure housing, language barriers, and uncertain legal status. Tackling these issues can foster broader social participation and improve occupational balance. Therefore, policy frameworks should integrate therapeutic models that position occupational balance as a driver of inclusive participation across various social domains. Such an approach aligns with the broader integration goals of host countries and provides a practical pathway to translate research into effective migration policy.

This study found a moderate negative correlation between occupational balance and perceived discrimination (group, personal, and total scores) among refugee participants. This result adds to the broader discourse on the structural determinants of health and inclusion in displaced populations by identifying discrimination as a significant barrier to achieving occupational balance. It underscores how experiences of discrimination can disrupt refugees’ daily activities and limit their ability to engage in meaningful roles. Our findings align with the previous literature suggesting that perceived discrimination may be a significant factor affecting refugees’ ability to maintain occupational balance [64,70]. For instance, Syrian refugees in Jordan experience occupational deprivation, emphasizing its impact on time use and occupational roles [64]. Two other significant factors associated with role change in the literature are social isolation and discrimination [35]. Similarly, Beiser et al. (2001) found that Southeast Asian refugees in Canada reported high levels of perceived discrimination, which negatively influenced their psychosocial well-being [70]. Given the persistence of discrimination across diverse host contexts, policy interventions should explicitly address systemic exclusion as part of occupational and social integration strategies.

The significant relationship between group discrimination and occupational balance in this study shows that refugees are affected not only by individual experiences but also by broader social-level discrimination. Drewski and Gerhards [71], for example, argue that Germany and Poland offer greater privileges to Ukrainian refugees compared to Syrians. Similarly, Deilamizade et al. [72] report that Afghan refugees face discrimination across multiple domains, including family, friendships, workplaces, neighborhoods, and healthcare settings. These cross-national disparities in treatment and access to resources reflect broader patterns of racialized hierarchies and selective humanitarianism within migration regimes. This underlines the need for policy frameworks that move beyond individual resilience narratives and instead address the collective dimensions of exclusion experienced by refugee groups. Although Turkish society initially demonstrated solidarity with Syrian refugees, concerns have been expressed about unemployment, increased crime rates, decreased access to public services, and the deterioration of national identity in response to the rapidly increasing refugee population [73]. These shifting public attitudes often influence government policy, contributing to the institutionalization of exclusionary practices that further restrict refugees’ participation and occupational balance.

The significant relationship between personal discrimination and occupational balance in this study plays a critical role in understanding how individual experiences shape the planning, maintenance, and adjustment of daily activities. This finding supports the view that discrimination affects not only psychological well-being but also disrupts functional aspects of everyday life. A study by Özaslan et al. [74] highlighted the negative effects of perceived personal discrimination on help-seeking behaviors in Syrian refugee adolescents. In addition, Baranik, Hurst and Eby [75] demonstrated that personal discrimination, in this case due to the status of being a refugee, resulted in higher levels of anxiety, depression and sleep disorders. These findings underscore how refugee status is not only a legal or political category but also a lived social identity that produces vulnerability through daily interpersonal interactions. This latter dimension remains underexplored in the context of migration research. Occupational therapists can support refugees’ mechanisms of coping with discrimination through community-based interventions, culturally sensitive activities, psychosocial support, vocational rehabilitation and advocacy. Integrating these strategies into broader refugee support systems can provide a model for practice-informed policy that promotes psychosocial resilience and active participation.

The regression analysis in this study showed that perceived discrimination did not directly influence occupational balance. This suggests that well-being and participation are the primary factors shaping occupational balance among refugees. The emergence of well-being and participation as significant predictors of occupational balance suggests that individuals may be experiencing discrimination in the background due to fear, past trauma, isolation, racism, or negative cultural beliefs [76]. However, the ability of some refugees to maintain occupational balance despite these experiences may point to the presence of protective factors. Previous research identifies social support, personal resilience, and cultural adaptation skills as critical in maintaining occupational balance [77,78]. For instance, Wood et al. [78] found that employment and volunteer work improved mental health and social integration among African refugees in Australia. Similarly, Markkanen [77] reported that refugee healthcare professionals in Finland were able to maintain occupational roles when given access to job opportunities, despite experiencing discrimination. These findings imply that focusing solely on perceived discrimination in vocational rehabilitation programs may not be sufficient. Instead, strengthening occupational balance requires enhancing refugees’ access to social support systems, developing their daily life skills, and supporting their cultural adaptation processes [79]. Empowerment-based approaches are especially recommended in occupational therapy to help refugees manage the effects and perceptions of discrimination [80]. Overall, the results of this study therefore underline that well-being and participation are the most significant predictive factors of occupational balance. We recommend that treatment, rehabilitation, and integration policies place these factors at their core to effectively support occupational balance in refugee populations.

## 5. Conclusions

This study investigated the predictors of occupational balance among adult refugees by examining the roles of personal well-being, participation, and perceived discrimination. The findings partially supported the research hypotheses: both personal well-being and participation significantly predicted occupational balance, while perceived discrimination showed no statistically significant association. These results highlight subjective well-being and social engagement as central predictive factors of occupational balance in the context of forced migration.

By focusing on occupational balance, a construct that remains underexplored in refugee health research, this study offers a novel contribution to the literature. The use of validated self-report instruments and the inclusion of psychosocial predictors provide a focused yet multidimensional perspective on how displaced individuals engage in meaningful daily activities. The findings also offer practical guidance for public health initiatives and NGO-led interventions aiming to enhance refugees’ occupational and social integration.

Several limitations should be acknowledged. First, recruiting participants through NGOs may have introduced selection bias, limiting the sample’s representativeness. Second, the short data collection period may have restricted the capture of temporal or situational variability. Third, reliance on self-administered questionnaires increased the risk of response bias. Fourth, the exclusive use of quantitative methodology may have overlooked qualitative nuances essential to fully understanding occupational experiences. Fifth, the statistical analysis relied solely on linear regression, without employing more advanced techniques such as structural equation modeling or moderation analysis.

Given these considerations, the R^2^ value of 0.244 reflects a modest yet acceptable level of explained variance. This is particularly relevant considering the complex and multifactorial nature of constructs such as occupational balance. In psychosocial and health research, R^2^ values below 0.30 are common and are still considered meaningful when supported by strong theoretical grounding and practical relevance.

Moreover, although our model assumes a unidirectional influence from well-being to occupational balance, the relationship may in fact be bidirectional. While previous studies often conceptualize well-being as a foundation for occupational balance, it is equally plausible that achieving occupational balance enhances psychological well-being. Since this study employed a cross-sectional design, we cannot draw causal conclusions. Future longitudinal or experimental research should investigate the directional nature of this relationship in greater depth.

Despite these limitations, this study provides theoretical and applied insights into the occupational lives of forcibly displaced individuals. It establishes a foundation for future interdisciplinary research linking occupational science, refugee health, and psychosocial adaptation. Future studies should consider longitudinal designs, qualitative methods, and broader ecological variables to deepen our understanding of occupational balance in vulnerable populations.

## Figures and Tables

**Table 1:** Descriptive statistics of participants (n = 260).

Categorical Variables	n	%
Gender		
Female	127	48.85
Male	133	51.15
Education Level		
Primary School	32	12.30
Middle School	39	15.00
High School	89	34.23
University	89	34.23
Master’s Degree	6	2.30
Doctorate	5	1.92
Employment Status		
Full time	115	44.23
Part time	31	11.92
Temporary	12	4.61
No employment	102	39.23
Marital Status		
Married	141	54.23
Single	97	37.30
Divorced	19	7.30
Widowed	3	1.15
**Numeric Variables**	**Min-Max**	**Mean ± SD**
Age	18–65	32.54 ± 1.48
OBQ11	7–33	17.37 ± 5.66
PWI-A	0–100	52.18 ± 16.84
PS	2–75	26.19 ± 13.49
Group Discrimination	4–20	12.37 ± 4.11
Personal Discrimination	4–20	10.48 ± 4.56
Total PDS	8–40	22.72 ± 7.73

Note: OBQ11: Occupational Balance Questionnaire; PWI-A: Personal Well-Being Index-Adult; PS: Participation Scale; PDS: Perceived Discrimination Scale; n: number; %: frequency.

**Table 2:** Pearson correlation coefficient results.

Variables	OBQ11	PWI-A	PS	Group Discrimination	Personal Discrimination
**Well-Being** PWI-A	0.421 **				
**Participation** PS	−0.357 **	−0.356 **			
**Perceived** **Discrimination** Group Discrimination Personal Discrimination Total PDS	−0.303 ** −0.227 ** −0.284 **	−0.345 ** −0.184 * −0.302 **	0.420 ** 0.342 ** 0.426 **	0.617 ** 0.878 **	0.898 **

Note. OBQ11: Occupational Balance Questionnaire; PWI-A: Personal Well-Being Index-Adult; PS: Participation Scale; PDS: Perceived Discrimination Scale. * *p* < 0.05, ** *p* < 0.01.

**Table 3:** Multiple linear regression analysis results.

Variable	*β*	SE	*t*-Value	*p*-Value
**Well-Being**				
PWI-A	0.323	0.021	5.327	<0.001 *
**Participation**				
PS	−0.197	0.026	−3.132	0.002 *
**Discrimination**				
Group Discrimination	−0.350	0.273	−1.761	0.080
Personal Discrimination	−0.354	0.269	−1.625	0.105
Total PDS	0.522	0.261	1.460	0.146

Note: *β* = standardized regression coefficient; SE = standard Error; *t*-value = test statistic for coefficient significance; PWI-A: Personal Well-Being Index; PS: Participation Scale; PDS; Perceived Discrimination Scale; * *p* < 0.05.

## Data Availability

Data are available from the corresponding author upon reasonable request.
